# G6PC3, ALDOA and CS induction accompanies mir-122 down-regulation in the mechanical asphyxia and can serve as hypoxia biomarkers

**DOI:** 10.18632/oncotarget.12931

**Published:** 2016-10-26

**Authors:** Yan Zeng, Yehui Lv, Li Tao, Jianlong Ma, Heng Zhang, Hongmei Xu, Bi Xiao, Qun Shi, Kaijun Ma, Long Chen

**Affiliations:** ^1^ Department of Forensic Medicine, School of Basic Medical Sciences, Fudan University, Shanghai, People's Republic of China; ^2^ Shanghai University of Medicine & Health Sciences, Shanghai, People's Republic of China; ^3^ Department of Physiology & Pathophysiology, School of Basic Medical Sciences, Fudan University, Shanghai, People's Republic of China; ^4^ Forensic Lab, Criminal Science and Technology Institute, Shanghai Public Security Bureau, Shanghai, People's Republic of China

**Keywords:** hypoxia, mechanical asphyxia, mir-122, G6PC3, ALDOA, Gerotarget

## Abstract

Hypoxia influences different cellular biological processes. To reveal the dynamics of hypoxia's effects on miRNA regulation *in vivo*, we examined the expression levels of all miRNAs in human brain and heart specimens from cases of mechanical asphyxia compared with those from cases of craniocerebral injury and hemorrhagic shock. We further validated differently expressed miRNAs in another 84 human specimens and rat models. We found that mir-122 was significantly down-regulated and that its putative targets *G6PC3*, *ALDOA* and *CS* were increased in the brain and cardiac tissues in cases of mechanical asphyxia compared with craniocerebral injury and hemorrhagic shock. Our data indicate that mir-122 and its targets *G6PC3*, *ALDOA* and *CS* play roles in the hypoxia responses that regulate glucose and energy metabolism and can serve as hypoxia biomarkers.

## INTRODUCTION

Hypoxia generally refers to insufficient oxygen supply to tissues that ranges from < 0.01% to 5%, and its duration can be chronic, acute or fluctuating. These variations may influence different cellular biological processes [1] such as the cell cycle [2], DNA damage repair [3] and mitochondrial metabolism [4]. For example, pO_2_ levels less than 10 to 15 mm Hg induce the expression of transcription factor HIF (hypoxia-inducible factor) and its downstream genes to maintain stable intracellular pH, glucose and angiogenesis levels. In addition, pO_2_ levels less than 10 mm Hg lead to decreases in adenosine triphosphate (ATP) and protein syntheses to reduce oxygen consumption, whereas extremely low pO_2_ levels of less than 1 mm Hg may cause apoptosis and a metabolic switch from oxidative phosphorylation to glycolysis to maintain adequate ATP levels [5]. Thus, the processes by which cells sense and respond to ambient oxygen concentrations are complex, and the highly coordinated metabolic response is critical in regulating the downstream effectors, such as HIF family transcription factors [6] and microRNAs (miRNAs) [7].

miRNAs are a class of small non-coding RNAs that regulate the stability and translation efficiency of their target mRNA by targeting the 3' untranslated region (UTR) [8, 9]. Emerging evidence indicates that miRNAs are involved in the metabolic regulation induced by hypoxia [10, 11]. And miRNA biogenesis is also influenced by hypoxia through HIF-dependent or HIF-independent transcriptional regulation [12].

Most previous studies of hypoxia have heavily relied on cell cultures and animal models; *in vivo* investigations, especially in humans, are lacking. We reason that a hypoxia shock environment is created in the body in cases of death by mechanical asphyxia and that the brain and heart are the tissues that are most sensitive to hypoxia. We first employed a microarray approach to examine the expression levels of all miRNAs in human brain and heart specimens from three mechanical asphyxia cases using two craniocerebral injury cases and two hemorrhagic shock cases as controls. In total, we validated 55 differentially expressed miRNAs using RT-qPCR in 48 brain and 36 heart specimens. We ultimately selected mir-122, which was expressed at a significantly low level in the both brain and heart specimens from mechanical asphyxia cases. We also reported reversed expression patterns of three predicted mir-122 target genes, *G6PC3*, *ALDOA* and *CS*, which encode metabolic enzymes, in the corresponding human specimens. These findings were confirmed in a rat hypoxia model. Our data suggest that mir-122 and its putative downstream target genes, *G6PC3*, *ALDOA* and *CS*, could serve as biomarkers for mechanical asphyxia and shed light on the pathogenesis of hypoxia in diseases.

## RESULTS

### We identified 71 differentially expressed mRNAs in brain specimens and 138 in cardiac specimens using microarrays

**Figure 1:**
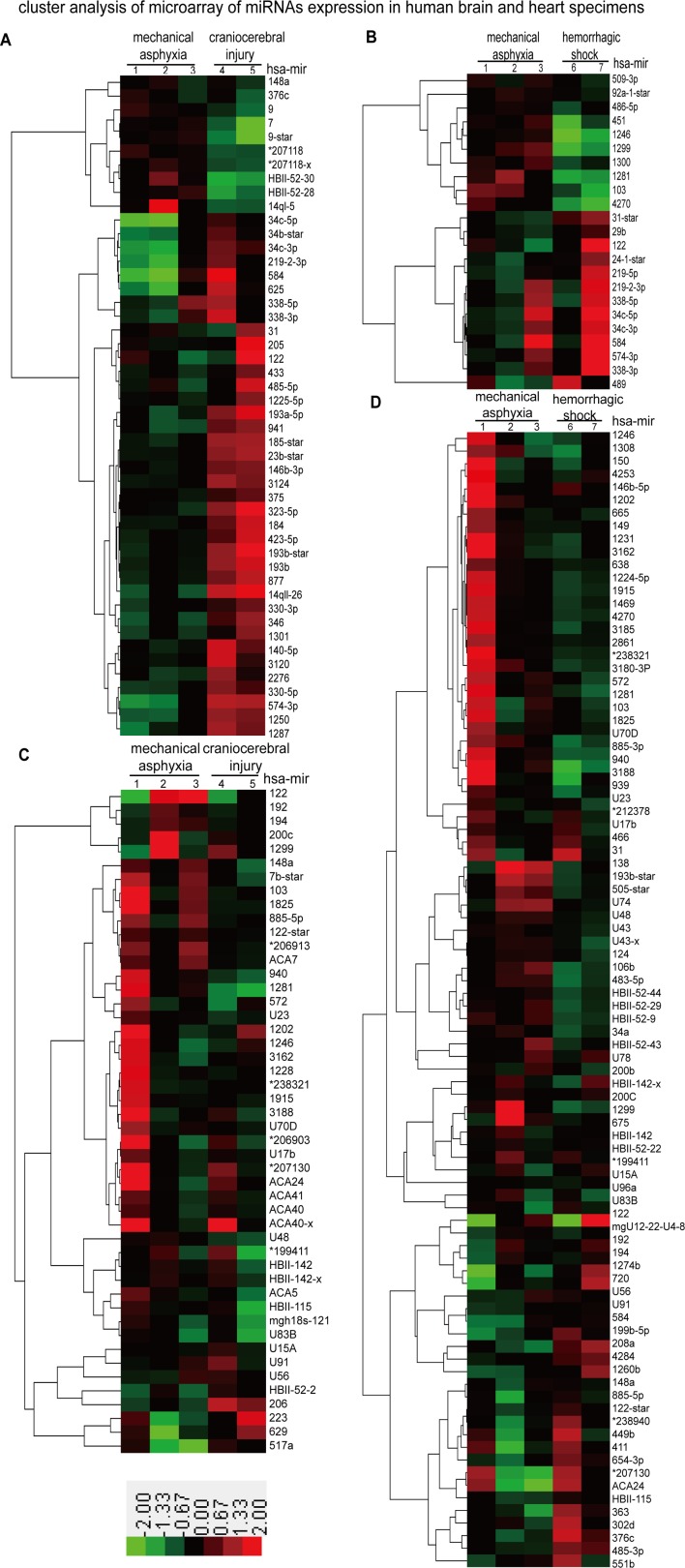
Microarray analyses of miRNA expression in human brain and heart specimens of three mechanical asphyxia cases, two craniocerebral injury cases and two hemorrhagic shock cases **A.** and **B.** Differentially expressed miRNA profiles of mechanical asphyxia cases compared with craniocerebral injury cases and hemorrhagic shock cases in human brain specimens; **C.** and **D.** Differentially expressed miRNA profiles of mechanical asphyxia cases compared with craniocerebral injury cases and hemorrhagic shock cases in human brain specimens. * = ENSG00000.

We hypothesized that hypoxic environments are created in the brain and heart immediately in mechanical asphyxia death and that alterations in miRNA expression levels may result. In this study, we detected the expression levels of the miRNAs in three mechanical asphyxia cases, two hemorrhagic shock cases and two craniocerebral injury cases using the Microarray 2.0 system. In total, 48 and 23 miRNAs differed in expression between the brain specimens of those who died of mechanical asphyxia compared with those who died from craniocerebral injury and hemorrhagic shock, respectively. Among these miRNAs, 10 out of 48 and 10 out of 23 were up-regulated by more than 2-fold, and others were down-regulated in the brain specimens that underwent mechanical asphyxia (Figure 1A, 1B and Table 1). Using the same approach, 48 and 90 miRNAs were differentially expressed in heart specimens, respectively. Among these miRNAs, 44 of 48 and 73 of 90 were up-regulated by more than 2-fold, and others were down-regulated in the cardiac specimens (Figure 1C, 1D and Table 1). Furthermore, eight miRNAs (mir-31, mir-122, mir-219-2-3p, etc.) in the brain specimens and sixteen miRNAs (mir-192, mir-148a, mir-122, etc.) in the cardiac specimens exhibited consistent changes as a result of mechanical asphyxia death compared with the other two causes of death (Table 1).

**Table 1:** Number of miRNAs that changed in brain and heart of mechanical asphyxia compared to controls

Specimens	Mechanical asphyxia comparing with control	Up/down-regulation	No. of regulated miRNA(fold change >2)	miRNAs regulated in both comparisons
Brain	Craniocerebral injury	up	10	mir-31; mir-122; mir-219-2-3p mir-34c-5p; mir-338-5p; mir-338-3p; mir-574-3p; mir-584
		down	38	
	Hemorrhagic shock	up	10	
		down	13	
Heart	Craniocerebral injury	up	44	mir-192; mir-148a; mir-122; mir-194; mir-572; mir-885-5p; mir-940; mir-1202; mir-1299; mir-1246; mir-1281; mir-103; mir-1825; mir-1915; mir-3162; mir-3188
		down	4	
	Hemorrhagic shock	up	73	
		down	17	

### RT-qPCR validation of microarray results and the robust reduction of mir-122 in mechanical asphyxia specimens

**Figure 2:**
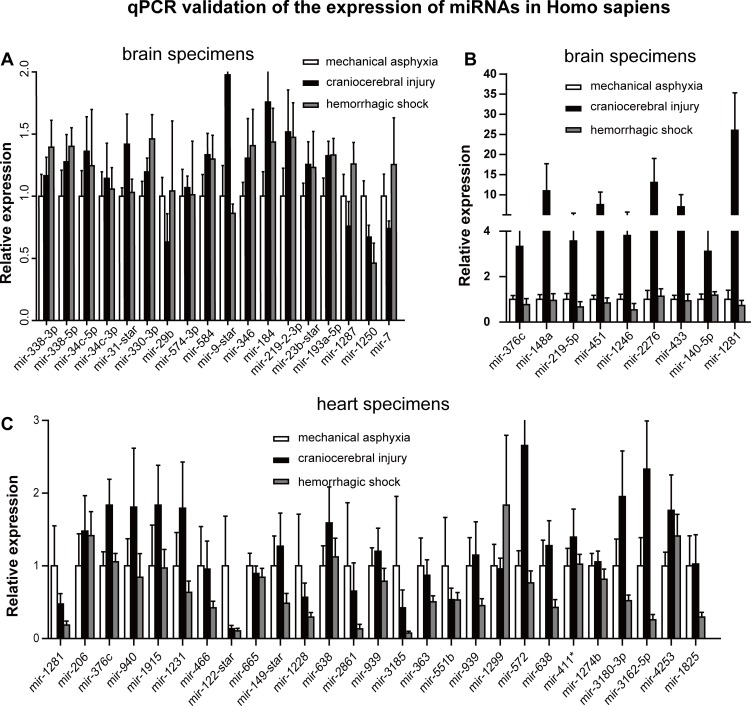
RT-qPCR detection of differentially expressed miRNA identified *via* microarray analyses in 48 human brain specimens and 36 human heart specimens from the indicated causes **A.** Comparison of miRNA expression levels in brain specimens; **B.** miRNAs up regulation in craniocerebral injury compared with the other two causes of death in brain specimens; **C.** Comparison of miRNA expression levels in heart specimens. MiRNA expression levels in mechanical asphyxia cases were normalized to 1, and the relative changes in the miRNA expression levels in craniocerebral injury cases and hemorrhagic shock cases were determined by comparisons with the expression levels in mechanical asphyxia cases.

We used U6 as a reference gene to determine that 55 of the abovementioned differentially expressed miRNAs exhibited greater changes *via* RT-qPCR and expanded our sample to 84 (48 brain and 36 cardiac) human specimens (Figure 2). We confirmed the expression changes (> 2-fold) of mir-184 and mir-1250 in brain and of mir-1281, mir-551b, mir-3185, mir-3162-5p and mir-1228 in heart in mechanical asphyxia cases (Figure 2A and 2C). Interestingly, we also noticed greater changes in several miRNAs (Figure 2B and 2C) in the brain or heart specimens of craniocerebral injury cases compared with hemorrhagic shock. The significance of these changes will be an interesting question for future investigations.

**Figure 3:**
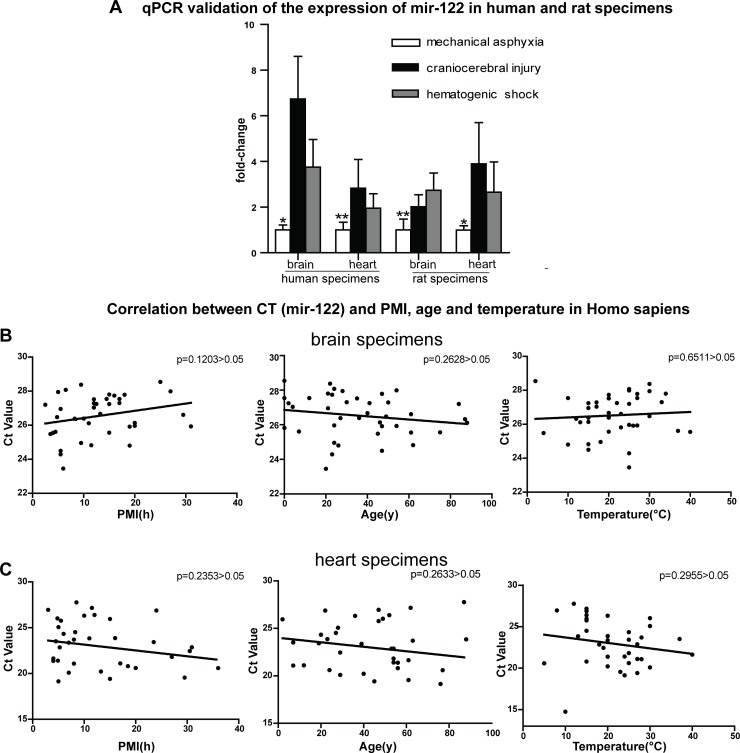
RT-qPCR validation of mir-122 expression in 48 human brain specimens, 36 heart specimens and 18 rats and analyses the relationships between the Ct values of mir-122 expression and postmortem of death, environmental temperature and age **A.** mir-122 down-regulation in both brain and cardiac tissues of human and rat from mechanical asphyxia cases compared with specimens from other cases. **B.** and **C.** Correlations between Ct values and postmortem interval (PMI), age and temperature in human brain samples and in human heart samples. *P* > 0.05 by the two-tailed *t*-test.

Further analysis of the RT-qPCR data from the 84 samples found that mir-122 was the most consistently down-regulated miRNA in response to mechanical asphyxia in both types of tissues comparing with the other two death causes. Specifically, 3.76- and 5.54-fold reductions in the mechanical asphyxia brain were noted compared with specimens from craniocerebral injury and hemorrhagic shock cases, respectively (Figure 3A). Regarding cardiac tissues, 2.83- and 1.96-fold reductions, respectively, were noted in the mechanical asphyxia cases (Figure 3A). Importantly, significant correlations with postmortem interval, environmental temperature and age were not observed for mir-122 expression (Figure 3B and Figure 3C), further indicating that mir-122 down-regulation is likely caused by hypoxia shock in mechanical asphyxia death.

### The glycometabolism-related genes *G6PC3, ALDOA*, and *CS* are putative targets of mir-122

**Figure 4:**
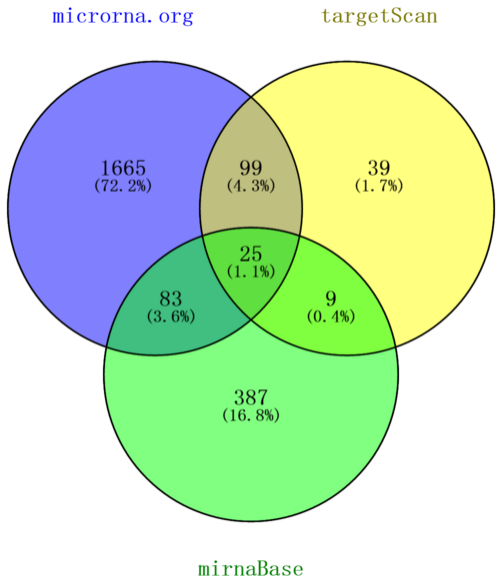
Venny analyses of mir-122 targets predicted by the microrna.org, TargetScan and MirTarBase databases

To elucidate the downstream mechanism of down-regulated mir-122 expression, we sought to identify mir-122 targets. To improve the accuracy of miRNA binding site prediction, we used three target prediction algorithms: microrna.org [13], TargetScan [14] and MirTarBase [15]. Venny analyses [16] revealed 25 genes that might be regulated by mir-122 (Figure 4). Using Gene Ontology (GO) analysis [17, 18], we observed significant enrichment in glycometabolism pathways. We further narrowed our analysis to *G6PC3* (glucose-6-phosphatase enzyme 3), *ALDOA* (fructose-1, 6-diphosphate aldolase A) and *CS* (citrate synthase), whose protein products are critical to glucose and energy metabolism (Table 2).

**Table 2:** Cluster of GO analysis of the 25 common predicted target genes

Pathway	*P* -value	Enrichment score	Cluster
Hexose metabolic process	2.7E-3	2.15	1
Monosaccharide metabolic process	4.0E-3		
Carbohydrate biosynthetic process	1.1E-2		
Glucose metabolic process	2.1E-2		
Phosphorus metabolic process	1.2E-2	1.29	2
Phosphate metabolic process	1.2E-2		
Response to organic substance	8.8E-2		
Phosphorylation	1.1E-2		
Protein amino acid phosphorylation	2.6E-1		
Regulation of phosphorylation	1.5E-1	0.81	3
Regulation of phosphorus metabolic process	1.6E-1		
Regulation of phosphate metabolic process	1.6E-1		
Response to organic substance	8.8E-2	0.62	4
Regulation of apoptosis	3.4E-1		
Regulation of programmed cell death	3.4E-1		
Regulation of cell death	3.4E-1		
Regulation of transcription, DNA- dependent	7.6E-1	0.11	5
Regulation of transcription	7.7E-1		
Regulation of RNA metabolic process	7.7E-1		
Transcription	8.4E-1		

### Overexpression of putative mir-122 downstream targets: *G6PC3, ALDOA*, and *CS*

**Figure 5:**
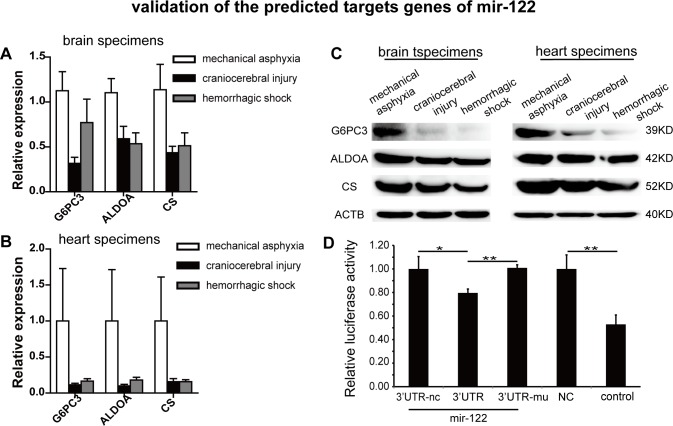
G6PC3, ALDOA and CS exhibited inverse correlations with mir-122 in specimens from the indicated causes of death and Dual luciferase reporter analysis of CS in 293T cells **A.** and **B.** Up-regulation of G6PC3, ALDOA and CS mRNA in human brain and heart specimens. **C.** Up-regulation of G6PC3, ALDOA and CS protein in rat brain and heart specimens. **D.** Dual luciferase reporter analysis of mir-122 and a reporter gene with predicted mir-122 target sequences (wildtype and mutant) in the CS 3'UTR in 293T cells.

It is generally accepted that miRNAs exert their function partly by down-regulating the expression of their target genes. We hypothesized that there are reverse correlations between mir-122 and *G6PC3*, *ALDOA* and *CS* mRNA levels. Indeed, we found that the mRNA levels of all three genes were significantly increased in the mechanical asphyxia specimens compared with the specimens from craniocerebral injury and hemorrhagic shock using the same set of 48 brain and 36 cardiac specimens described above (Figure 5A and 5B). Consistently, mir-122 and protein level analyses from the three cause-of-death models in rats (*n* = 6) revealed the same trend (Figure 3A and Figure 5C). We further confirmed that mir-122 overexpression suppresses the expression of a luciferase reporter gene containing the putative wild-type but not the mutated mir-122 target sequence from *CS* 3'UTR in human 293T cells (Figure 5D). Evidence of G6PC3 and ALDOA as putative targets has been confirmed using luciferase reporter [19]. Our data coordinate with prevails studies indicating that mir-122 regulates the expression of the mRNAs and proteins related to G6PC3 [20], ALDOA [21] and CS [22] in cell cultures.

## DISCUSSION

Microarray analyses of brain and cardiac specimens from the seven human cases, which included three cases of death by mechanical asphyxia, two of death by craniocerebral injury and two of death by hemorrhagic shock, revealed 71 differentially expressed miRNAs in the brain and 138 in the heart. Further RT-qPCR validation of miRNAs in both organs in a larger sample size of 48 brain and 36 cardiac specimens with the same three causes of death found that several miRNAs were differentially expressed in the mechanical asphyxia specimens. Specifically, mir-122 expression was significantly reduced in the brains and cardiac tissues of mechanical asphyxia cases compared with the specimens from craniocerebral injury and hemorrhagic shock cases. We also observed reversed expression patterns of the three predicted miR-122 targets, *G6PC3, ALDOA* and *CS*, which encode glycometabolic enzymes, in the corresponding specimens. These findings were validated in a rat hypoxia model, further confirming their biological relevance.

Hypoxia occurs during and after death, and visceral organs such as the brain and heart will experience hypoxia regardless of cause of the death. In forensic science, the distinguishing feature of the death by mechanical asphyxia is the disruption of oxygen intake by force [23]. The body experiences a period of struggle, and the organism suffers from hypoxia longer and more intensely than in other cause of death. A hypoxia condition can induce the secretion of surfactant protein-A (SP-A). And an increase of SP-A in mechanical asphyxia cases demonstrates that hypoxia is the distinguishing feature of death in such cases [24]. In addition, Cecchi et al. have demonstrated that HIF-1a (hypoxia-induced factor 1-a), a target of mir-122 expressed in response to hypoxia, was up-regulated in mechanical asphyxia compared with craniocerebral injury, natural death and other causes of death [25, 26]. Our findings thus indicate that mir-122 reduction might be the response to hypoxia in mechanical asphyxia.

We observed significantly increased *G6PC3*, *ALDOA* and *CS* expression accompanied by reductions in mir-122 expression in mechanical asphyxia specimens, indicating an elevated glucose demand under acute hypoxia conditions. Kyoungsub et al. found that mir-122 decreased and activated glycolytic metabolism with low ATP synthesis in hepatocellular carcinoma cancer stem cells in a hypoxia microenvironment [27]. Additionally, to compensate for the reduction in ATP production under hypoxic conditions, cancer cells can stimulate glucose uptake and metabolism by inducing GLUT3 and ALDOA [28, 29]. ALDOA is a glycolytic enzyme that can up-regulated by inhibiting mir-122 in the liver [21, 30]. Finally, Miranda et al. have demonstrated that mir-122 is down-regulated intracellularly with the excessive glucose spared from glycolysis going towards storage in breast cancer cells. They also found that mir-122 is up-regulated in cancer-secreted enclosed vesicles and transfers to normal cells to suppress CS expression and glucose utilization in these cells [22]. Furthermore, G6PC3 is a gluconeogenic enzyme that can be stimulated by mir-122 reduction to contribute to gluconeogenesis [20, 31]. All of these studies demonstrate that mir-122 is down-regulated in hypoxia and can stimulate intracellular glucose by up-regulating G6PC3 and ALDOA regardless of whether it is stimulated by glycolysis or by gluconeogenic factors. It also increases glucose utilization by up-regulating ALDOA and CS to sustain ATP production for cell life. In this study, the elevated expression of G6PC3, ALDOA and CS, which influence glucose and energy metabolism, may play important roles in the hypoxia response caused by mechanical asphyxia.

Our findings indicate that an acute metabolic response to hypoxia occurs in human bodies in cases of mechanical asphyxia and is likely regulated by mir-122. As the first human report using specimens from cases of mechanical asphyxia, we anticipate that our findings will not only provide biomarkers for mechanical asphyxia but also shed light on the *in vivo* understanding of the pathogenesis of the many types of hypoxia involved in diseases.

## MATERIALS AND METHODS

### Biological materials

#### Human specimens

Ethical approval for the use of human samples was obtained from the Science and Ethics Committee of Fudan University. We obtained 98 brain and cardiac samples from the Forensic Lab of the Shanghai Public Security Bureau with their written informed consent. All samples were assigned to one of three groups according to the cause of death: mechanical asphyxia, craniocerebral injury or hemorrhagic shock. The details of the samples are provided in Table 3.

**Table 3:** Information of human samples

Group	*n*	Average Age(y) at death	Average PMI(h)	Average temperature(°C) at death	Gender M/F
**Mechanical Asphyxia**	21	38	15	19	17/4
**brain trauma**	18	38	16	23	15/3
**Hemorrhagic Shock**	18	36	10	20	14/4

#### Animal specimens

Rats were purchased from the Department of Animal Science Laboratory of Fudan University. In total, 18 male Sprague-Dawley rats (body weight 220 ± 20 g) were randomly assigned to three groups (mechanical asphyxia, craniocerebral injury and hemorrhagic shock) to collect the anterior region of the brain and the cardiac muscle of the apex cordis. All samples were placed in RNA Later solution (Takara, Japan) immediately after collection. The animal experiments described in the study were performed in accordance with the principles for the Care and Use of Laboratory Animals and were approved by the Science and Ethics Committee of Fudan University. In the mechanical asphyxia death group, a nylon rope (diameter 3 mm) was used to create a sliding loop that was fixed to the top of a bar, and the rats were placed in the loop, causing suffocation death based on their own gravity. In the craniocerebral injury death group, a 10-g weight fell freely from a 20-cm height within the vertical catheter and hit the dura. In the hemorrhagic shock death group, the bilateral carotid artery was isolated and cut to induce death *via* shock.

#### RNA purification and reverse transcription

Total RNA was extracted from the samples following the methods described by *Ma et al.* [32]. Briefly, cDNA was generated using a PrimeScript RT Reagent Kit (Takara, Japan) according to the manufacturer's protocol, and 500 ng of total RNA was reverse transcribed by adding a polyA tail using a One-Step Prime-Script miRNA cDNA Synthesis Kit (Takara, Japan) according to the manufacturer's protocol for microRNA analysis. The cDNA product was then diluted by a ratio of 1:10 for further use and was stored at −20°C for RT-qPCR.

### Microarray and analysis

The RNA samples were diluted to 100 ng/μl and independently used to perform the target preparation using a whole-transcript sense target labeling protocol (Affymetrix, High Wycombe, UK). Cluster analysis was performed using the unweighted pair group method, with arithmetic means based on Euclidean distance and with hierarchical clustering applied to the normalized data. Fold changes were calculated to identify the different expression profiles of mechanical asphyxia, craniocerebral injury and hemorrhagic shock. We used TreeView software to visualize the cluster analysis results.

### Gene expression quantification (qRT-PCR assay)

To test the candidate miRNAs acquired from the microarray analysis, qRT-PCR was performed using the miRNA qRT-PCR SYBR Kit (Takara, Japan) in a final reaction volume of 20 μl. The reaction mix was run on an ABI Prism 7500 fluorescence quantitative PCR instrument (Applied Biosystems, USA) according to the manufacturer's protocol. The results were normalized to U6 levels. Primer details are provided in Table 4. The primers were synthesized by Sangon Biotech China, Shanghai.

**Table 4:** Primers used to amplify RNA markers by RT-qPCR

Genes	Sequence of primer	
	F-primer	R-primer
hsa-mir-122	GCGTGGAGTGTGACAATGGTG	Uni-miR qPCR Primer
rno-mir-122	TGGAGTGTGACAATGGTGTTTG	Uni-miR qPCR Primer
has/rno-U6	TGACACGCAAATTCGTGAAGCGTTC	Uni-miR qPCR Primer
hsa-G6PC3	GATGCCTAGCCTGGCTTATT	CAGGACAGCGCCAGTTATTA
hsa-ALDOA	GCGTTGTGTGCTGAAGATTG	GCTGGCAGATACTGGCATAA
hsa-CS	CATCCGTTTCCGAGGCTTTA	CCTGTTCCTCTGTTGGGATATG
hsa-18s	GCCATGCATGTCTGAGTACGC	CCGTCGGCATGTATTAGCTC

### Target prediction

To select plausible targets to validate the significance of the detected miRNAs, microrna.org, TargetScan, and MirTarBase were used. Given that miRNAs may have a multitude of conserved miRNA species, UTR pairs were used.

### Western blot analyses

Total proteins from the brain and myocardium tissues of rats were extracted using RIPA protein lysis buffer with phenylmethylsulfonyl fluoride (PMSF), size-fractionated on 10% SDS-PAGE gels and transferred onto 0.45-um polyvinylidene difluoride (PVDF) membranes (Millipore, USA) according to the standard protocol. The following primary antibodies were used: anti-ALDOA (Abcam, ab169544, USA), anti-CS (Abcam, ab96600, USA), anti-G6PC3 (Biorbyt, orb156993, United Kingdom), and anti-ACTB (Santa Cruz Biotechnology). The secondary antibody was horseradish peroxidase conjugated to goat anti-rabbit IgG (sc-2004, Santa Cruz Biotechnology). Luminous detection was performed using ECL Western Blotting Substrate (Thermo Fisher, USA), and the signal was quantified using NIH ImageJ and normalized to ACTB.

### 3'UTR reporter assay

The 3'UTR fragment of the candidate target gene was subcloned into the Xba I site downstream of the luciferase gene in the pGL3-control vector (Promega). The negative controls were 3'UTR-mu and c-GFP. HEK293T cells were infected with c-GFP and mir-122 or mir-122M for 24 h. The cells were then seeded into 24-well plates and co-transfected with 0.5 μg of the respective pGL3-3'UTR construct and 0.05 μg of the pGL-TK vector (Promega). After 48 h, luciferase activity was measured using a Dual-Luciferase Reporter Assay System Kit (Promega).

### Statistical analysis

To calculate the relative expression levels of the target miRNAs, we performed ΔCt normalization. Unpaired *T*-tests (with significance levels of *p* < 0.05) were calculated for the mechanical asphyxia, brain trauma and hemorrhagic shock conditions. Statistical analysis was performed using SPSS 17.0, and the data are presented with GraphPad Prism 5.0 software. The results were considered statistically significant when *p* < 0.05.
